# Device infection in patients undergoing pacemaker or defibrillator surgery: risk stratification using the PADIT score

**DOI:** 10.1007/s10840-024-01759-1

**Published:** 2024-01-30

**Authors:** John de Heide, Marisa van der Graaf, Marijn J. Holl, Mark G. Hoogendijk, Rohit E. Bhagwandien, Sip A. Wijchers, Dominic A. M. J. Theuns, Tamas Szili-Torok, Felix Zijlstra, Mattie J. Lenzen, Sing-Chien Yap

**Affiliations:** Department of Cardiology, Thorax Center, Cardiovascular Institute, Erasmus MC, Dr. Molewaterplein 40, 3015 GD Rotterdam, the Netherlands

**Keywords:** Cardiac implantable electronic device, Endocarditis, Implantable cardioverter-defibrillator, Infection, Pacemaker, Pocket infection

## Abstract

**Background:**

The use of an antibacterial envelope is cost-effective for patients at high risk of developing cardiac implantable electronic device (CIED) infection. The identification of these high-risk patients may be facilitated using a clinical risk score. The aim of the current study is to evaluate the PADIT score for identifying high-risk patients in patients undergoing a CIED procedure in a tertiary academic center.

**Methods:**

This was a retrospective single-center study of consecutive patients undergoing a CIED procedure between January 2016 and November 2021. Patients who received an antibacterial envelope were excluded from this study. The primary endpoint was hospitalization for a CIED infection in the first year after the procedure.

**Results:**

A total of 2333 CIED procedures were performed in the study period (mean age 61.6 ± 16.3 years, male sex 64.5%, previous CIED infection 1.7%, immunocompromised 5.4%). The median PADIT score was 4 (interquartile range, 2–6). CIED infection occurred in 10 patients (0.43%). The PADIT score had good discrimination in predicting major CIED infection (*C*-statistic 0.70; 95% confidence interval [CI] 0.54 to 0.86, *P* = 0.03). Using an optimal PADIT score cut-off value of 7, the risk of CIED infection was higher in the patients with a PADIT score of ≥ 7 in comparison to those with a lower PADIT score (1.23% vs. 0.26%, *P* = 0.02; odds ratio 4.8, 95% CI 1.4 to 16.6, *P* = 0.01).

**Conclusions:**

The PADIT score is a clinically useful score for identifying patients at high risk of developing CIED infection. The use of an antibacterial envelope in these high-risk patients may be cost-effective.

**Graphical Abstract:**

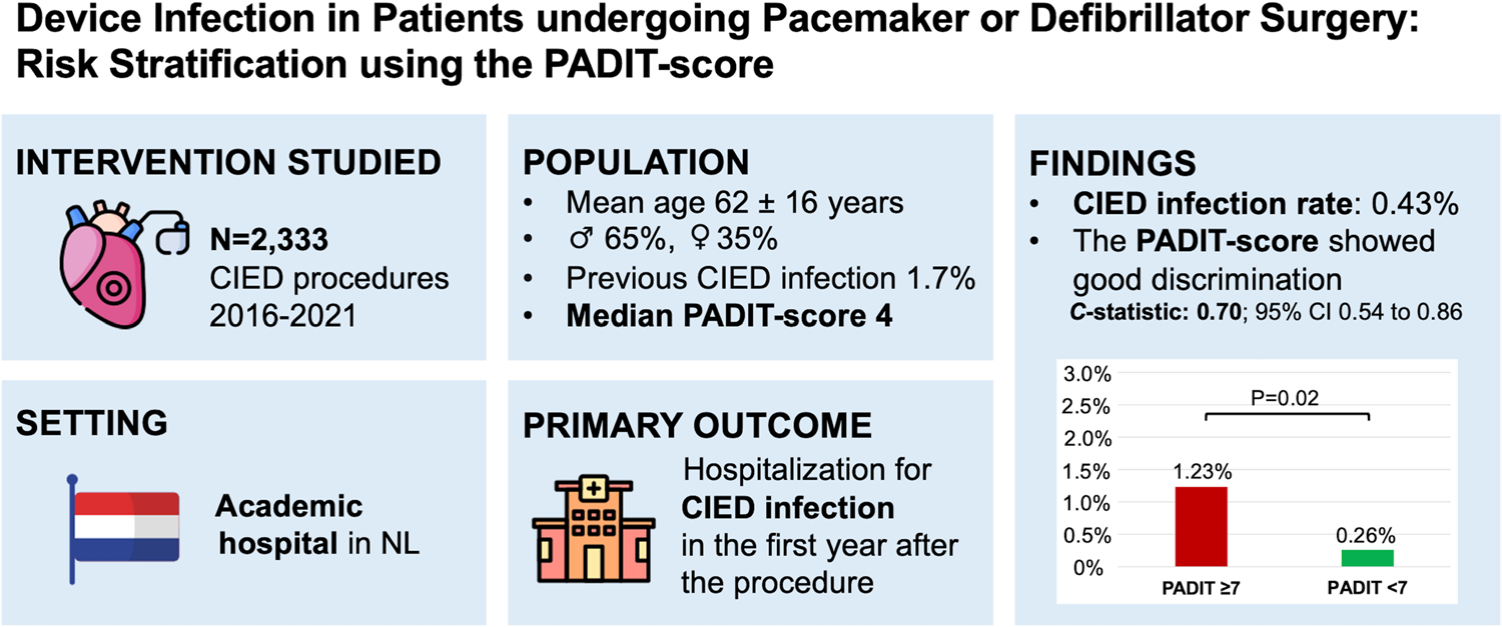

**Supplementary Information:**

The online version contains supplementary material available at 10.1007/s10840-024-01759-1.

## Introduction

The risk of device infection is approximately 1% in the first year after cardiac implantable electronic device (CIED) implantation [1–4]. CIED infection is associated with substantial morbidity and increased mortality risk. Patients with CIED infection often require hospitalization, prolonged antibiotic treatment, timely removal of their CIED system, and often CIED reimplantation [5, 6]. Management of CIED infection is therefore associated with a high financial health care burden [7]. Mitigation of the risk of CIED infection is crucial and preventive measures include among others preoperative antibiotics, chlorhexidine skin preparation, and avoidance of heparin bridging [8–10]. In 2019, the WRAP-IT (World-wide Randomized Antibiotic Envelope Infection Prevention) trial demonstrated that an absorbable antibacterial envelope reduced the risk of major CIED infection by 40% in patients undergoing CIED reoperations and initial cardiac resynchronization therapy defibrillator (CRT-D) implantation [11]. Cost-effectiveness studies demonstrated that an antibacterial envelope had the most favorable cost-effectiveness profile in high-risk patients [7, 12]. An antibacterial envelope is thus recommended by the European Heart Rhythm Association (EHRA) in high-risk patients [13]. The identification of high-risk patients may be aided using risk calculators such as the PADIT (Prevention of Arrhythmia Device Infection Trial) score which uses five independent clinical and procedural predictors of CIED infection [14, 15]. The aim of the present study is to evaluate the usefulness of the PADIT score in identifying patients at high risk for CIED infection in a tertiary academic center.

## Methods

### Study cohort

We retrospectively evaluated all consecutive adult patients who underwent a pacemaker or defibrillator surgery between January 2016 and November 2021 in our academic center. Exclusion criteria were a recent (< 3 months) transvenous lead extraction, implantation of a leadless pacemaker, and use of an anti-bacterial envelope. The antibacterial envelope is only sparsely used in the Netherlands considering the lack of reimbursement. Data were collected from the electronic medical records. Our center is a high-volume tertiary center with approximately 430 implants annually and is a referral center for heart transplantation, left ventricular assist devices, adult congenital heart disease, lead extraction, inherited cardiac disease, and pediatric cardiac surgery.

### Anticoagulation regimen

Patients using direct oral anticoagulants (DOACs) discontinued their drug 24–48 h before surgery depending on their renal function. All DOACs were restarted 24 h after the end of surgery, unless stated otherwise by the operator. In patients using vitamin K antagonists (VKA), the target international normalized ratio was 2.0 to 2.5 in the morning of the procedure. Patients with continued VKA usually attained to their regular dosing schedule. Heparin bridging was avoided if possible.

### Antibiotic treatment regimen

All patients received systemic antibiotic prophylaxis within 1 h of the procedure. This was either a single dose of intravenous cefazolin (2 g) or intravenous clindamycin (600–900 mg depending on weight) if patients were allergic to beta-lactam antibiotics (i.e., penicillin, cephalosporins). Vancomycin was reserved for patients with an allergy to both cefazoline and clindamycin. This local antibiotic regimen was based on the national guidelines for antibiotic use in the Netherlands. We postponed CIED procedures in patients who had a fever or high C-reactive protein at the day of their surgery. No postoperative antibiotic therapy was routinely given.

### Peri-procedural setting

CIED procedures were performed in a catheterization lab which is a sterile environment which complies with the requirements of an operating room Class 2 according to the Dutch Infection Prevention Taskforce guidelines. This includes the use of two semi-restricted zones and tightly controlled ranges for temperature, pressure (i.e., positive pressure of at least 5 Pa from zone A to B), relative humidity, and ventilation rates (i.e., minimum of 10 total air exchanges per hour, use of air filter using HEPA). The number of staff was kept to a minimum and usually consisted of a physician, scrub nurse, circulating nurse, and a CIED technician. All procedures were performed or supervised by an EHRA-certified cardiac device specialist with a large experience in CIED implantations. CIED procedures were also performed by fellows. After a surgical scrub, the operator(s) and scrub nurse wore a sterile gown, cap, mask, and non-powdered double gloves. The scrub nurse performed the prepping and draping. The skin was prepared with antiseptic formulated with 0.5% chlorhexidine digluconate and 70% alcohol and sufficient time was given to allow the antiseptic preparation to dry. After the application of sterile drapes, the operating field was covered by an adhesive iodophor-impregnated incise drape, except in patients who were allergic to iodine. For transvenous lead implantation, the primary choice for venous access was the cephalic vein. For generator replacements and upgrade/revision procedures, we used a pulsed electron avalanche knife (PEAK) PlasmaBlade™ (Medtronic, Minneapolis, MN, USA). This is an electrocautery device which uses pulses of radiofrequency energy to cut and coagulate soft tissue without the thermal damage to surrounding tissues normally seen with traditional electrosurgery. Meticulous attention was paid to hemostasis before wound closure in several layers. The final skin closure was performed with an absorbable suture. A sterile dressing was applied to the wound for a minimum of 4 days. Pressure dressing was only applied in selected patients (e.g., oozing of wound). Patients were instructed to keep the wound dry for a minimum of 4 days. The peri-procedural measures are largely in line with the current EHRA consensus document [16].

### Discharge and follow-up

Patients undergoing a generator replacement only were discharged on the same day of the procedure after clinically significant pocket hematoma had been ruled out. Patients undergoing a *de novo* device implantation, upgrade, or revision were discharged the day after the procedure. On the day of discharge, these patients underwent a physical examination of their device pocket, a device interrogation, a chest X-ray (to rule out pneumothorax and lead dislodgement), and a bed-side echocardiogram (to rule out pericardial effusion). Two weeks after discharge the patients were seen at the outpatient clinic for wound inspection and device interrogation. Thereafter, device interrogation was performed every 6 months with or without remote monitoring. Every 3 months a CIED complication meeting was organized in which all CIED-related complications, including infections, are discussed by the operators. Furthermore, our center organizes a weekly regional multidisciplinary Endocarditis Heart Team in which patients with suspected endocarditis, including CIED-related infections, are discussed. Finally, our center is the only center in the region which performs transvenous lead extractions and is a high-volume center for transvenous lead extractions (approximately 50 cases annually).

### PADIT score

The PADIT score was developed to predict the risk of hospitalization for device infection within 1 year [14]. A correction was published to the original risk score and this modified score was used [15]. This model includes 5 independent predictors of CIED infection including number of Prior procedures, Age, Depressed renal function (estimated glomerular filtration rate [GFR] < 30 mL/min), being Immunocompromised, and procedure Type. Immunocompromised was defined in the PADIT trial as receiving therapy that suppresses resistance to infection (e.g., immunosuppression, chemotherapy, radiation, long-term, or recent high-dose steroids) or having a disease that is sufficiently advanced to suppress resistance to infection (e.g., leukemia, lymphoma, HIV infection). The minimum risk score is 0 and the maximum is 13 (Supplemental Table 1). The PADIT score was calculated using the online calculator (https://padit-calculator.ca) which used the corrected version of the PADIT score. Based on the PADIT score, 3 risk categories could be identified according to the original publication: low risk (≤ 4), intermediate risk [5, 6], and high risk (≥ 7) [14].

### Study endpoint

The primary endpoint was a CIED infection requiring hospitalization within 1 year of the procedure. This definition was also used in the original PADIT trial (2). The diagnosis of CIED or pocket infection followed the 2019 International CIED Infection criteria [16].

### Statistical analysis

Continuous parameters were tested for normality before analysis and are expressed as mean ± standard deviation (SD) or median (interquartile range [IQR]), as appropriate. Categorical data are presented as frequencies and percentages. Comparisons between groups were performed with an independent Student *t*-test, chi-square test, Fisher exact test, or a Mann–Whitney *U* test, as appropriate. We used the receiver operating characteristic (ROC) curve to evaluate the performance of the PADIT score to predict the 1-year risk of device infection. Discrimination was assessed by using the Harrell’s *C*-statistic. Model discrimination was deemed poor if the *C*-statistic was between 0.50 and 0.70, good between 0.70 and 0.80, and excellent if > 0.80. Binary logistic regression analysis was performed to test the diagnostic properties of the optimal PADIT score threshold. Odds ratios will be presented with their corresponding 95% confidence intervals (CI). All analyses were two-tailed; a *P*-value < 0.05 was considered statistically significant. Statistical analyses were performed using SPSS software (SPSS, version 28.0.1.0; IBM, Chicago, IL).

## Results

### Study population

**Fig. 1:**
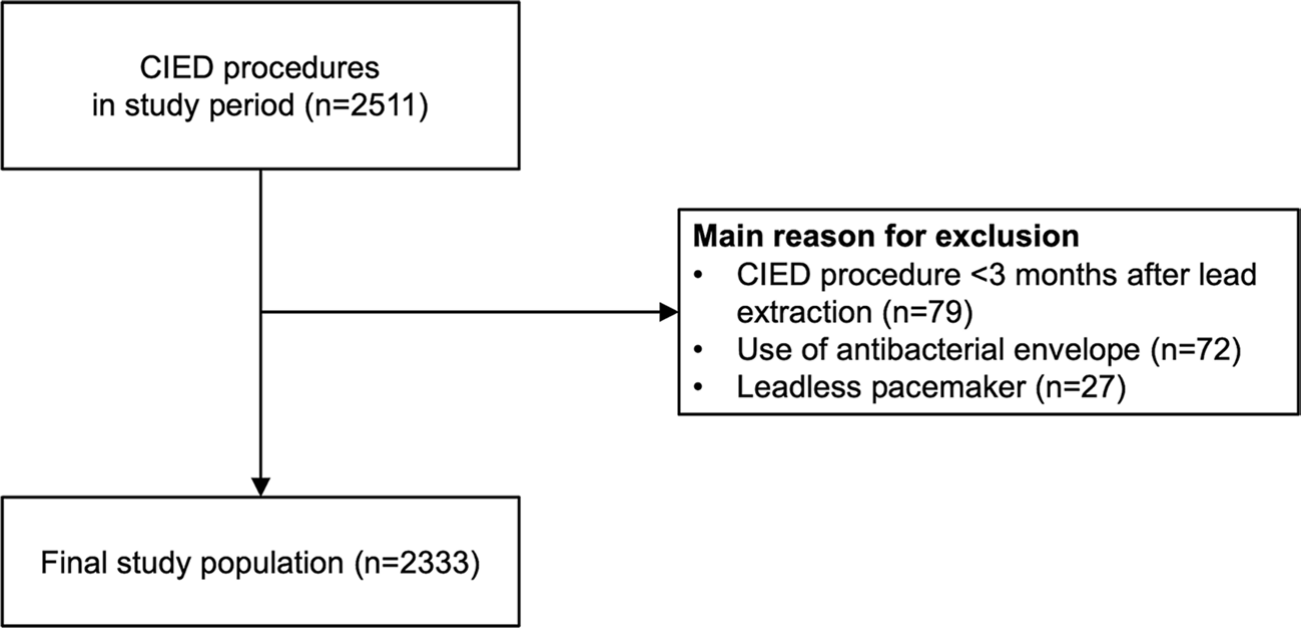
Flow chart study population. Abbreviations: CIED, cardiac implantable electronic device

A total of 2511 CIED procedures were performed during the study period. After the exclusion of patients who did not fulfil the eligibility criteria, the final study population consisted of 2333 CIED procedures in 2105 patients (Fig. 1). Baseline characteristics of the study population are presented in Table 1. The mean age at the time of the procedure was 61.6 ± 16.3 years and 64.5% were male. A previous CIED infection was present in 1.7%, and chronic kidney disease stage IV to V (eGFR < 30 ml/min) was present in 5.4%. One hundred twenty-seven patients (5.4%) were immunocompromised. The most common procedure was an ICD procedure (new or generator replacement, 42.6%), followed by a pacemaker procedure (new or generator replacement, 29.3%), CRT procedure (new or generator replacement, 16.6%), and revision and/or upgrade procedure (11.5%). A total of 1117 patients (47.9%) would be considered potential WRAP-IT patients (i.e., CIED reoperations and initial CRT-D implantation).

**Table 1:** Patient and procedural characteristics

Characteristic	Total (*n* = 2333)
Age (years)	61.6 ± 16.3
Male sex	1505 (64.5)
Body mass index	23.4 ± 4.5
PADIT score	4 (2–6)
Medical history	
Chronic heart failure	1028 (44.1)
Non-ischemic cardiomyopathy	680 (29.1)
Ischemic cardiomyopathy	497 (21.3)
History of atrial fibrillation	819 (35.1)
Coronary artery disease	847 (36.3)
CKD stage III to V (eGFR < 60 mL/min)	714 (30.6)
CKD stage IV to V (eGFR < 30 mL/min)	126 (5.4)
eGFR (mL/min)	69 ± 24
Hypertension	773 (33.1)
Diabetes mellitus	415 (17.8)
COPD	211 (9.0)
History of stroke	196 (8.4)
History of transient ischemic attack	171 (7.3)
Peripheral artery disease	155 (6.6)
History of bleeding	119 (5.1)
Mechanical heart valve	113 (4.8)
History of CIED infection	40 (1.7)
Immunocompromised	127 (5.4)
Antithrombotic therapy	
Vitamin K antagonist	871 (37.3)
Antiplatelet agent	729 (31.2)
Direct acting oral anticoagulant	355 (15.2)
Type of procedure	
New pacemaker (excluding CRT)	548 (23.5)
New transvenous ICD (excluding CRT)	436 (18.7)
New subcutaneous ICD	178 (7.6)
New CRT pacemaker	54 (2.3)
New CRT defibrillator*	136 (5.8)
Pacemaker generator replacement (excluding CRT)*	135 (5.8)
ICD generator replacement (excluding CRT)*	380 (16.3)
CRT generator replacement*	198 (8.5)
Revision or upgrade procedure*	268 (11.5)
Subpectoral position	148 (6.3)
Fellow participation in procedure	1238 (53.1)
Procedure duration (min), median (IQR)	60 (40–84)

Data depicted as (*n*, %), mean ± standard deviation, or median (IQR)

*ACEI* Angiotensin-converting enzyme inhibitor; *ARB* Angiotensin receptor blocker; *CIED* Cardiac implantable electronic device; *CKD* Chronic kidney disease; *COPD* Chronic obstructive pulmonary disease; *CRT* Cardiac resynchronization therapy; *eGFR* Estimated glomerular filtration rate; *ICD* Implantable cardioverter-defibrillator

*Potential WRAP-IT candidate

**Fig. 2:**
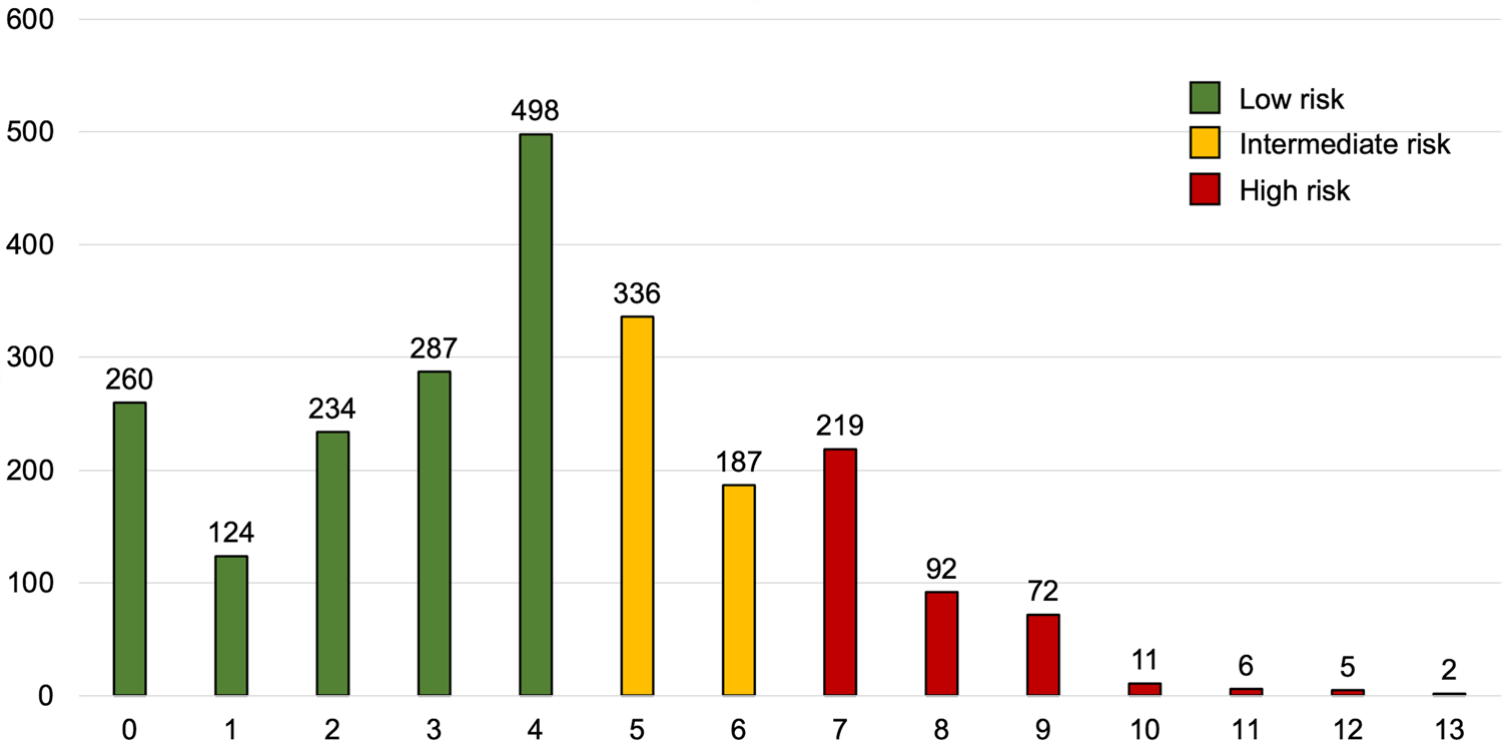
Distribution of PADIT score in the study population. PADIT score classified as low (≤ 4), intermediate (5-6), or high risk (≥ 7) in accordance with the original paper by Birnie et al. [14]

The median PADIT score was 4 (IQR, 2–6). Figure 2 shows the distribution of the PADIT score in the study population. The proportion of patients with low (≤ 4 points), intermediate (5–6 points), and high-risk PADIT score (≥ 7 points) was 1403 (60.1%), 523 (22.4%), and 407 (17.4%), respectively.

### Primary endpoint and PADIT score

Within 1 year of follow-up, hospitalization for CIED infection occurred in 10 patients (0.43%, 95% CI 0.21–0.79%). Details regarding the CIED infections are summarized in Table 2. Most cases occurred within the first 5 weeks after the procedure (80%) and *Staphylococcus aureus* was the most frequently isolated pathogen (40%). Almost all patients had complete removal of their CIED system (90%).

**Table 2:** Details of CIED infection requiring hospitalization

Case	Age/sex	PADIT score	Index procedure	CIED infection	Timing (days after procedure)	Causative pathogen	Management
1	45/F	5	Pacemaker implantation	Pocket infection	4	-	Complete system removal, cefuroxime iv, reimplantation single chamber pacemaker contralateral side
2	34/F	7	ICD generator change (no CRT)	Pocket infection	13	*CNS*	Complete system removal with TLE, flucloxacillin iv, reimplantation subcutaneous ICD
3	65/F	4	ICD implantation	Pocket infection	20	*S. aureus*	Clindamycin oral, pocket revision with Tyrx envelope
4	69/M	9	CRT-D generator change	Pocket infection	21	*S. epidermidis*	Complete system removal with TLE, no antibiotics, no reimplantation due to LVEF 49%
5	55/M	4	ICD implantation	Systemic CIED infection	24	*S. aureus*	Complete system removal, flucloxacillin iv, reimplantation dual chamber ICD after antibiotic treatment
6	42/F	2	Pacemaker implantation	Systemic CIED infection	29	*S. aureus*	Complete system removal, flucloxacillin iv, reimplantation dual chamber pacemaker contralateral side
7	72/M	8	CRT-D generator change	Pocket infection	31	*S. aureus*	Complete system removal with TLE, flucloxacillin oral, reimplantation CRT-D with antibiotic envelope contralateral side
8	19/M	4	ICD implantation	Pocket infection	33	*K. variicola*	Complete system removal, no antibiotics, reimplantation dual chamber ICD contralateral side
9	65/F	7	ICD generator change (no CRT)	Systemic CIED infection	146	*E. faecalis*	Complete system removal with TLE, amoxicillin iv, ceftriaxone iv, reimplantation subcutaneous ICD
10	65/M	9	Upgrade to ICD	Systemic CIED infection	260	*E. faecalis*	Complete system removal with TLE, vancomycin iv, no reimplantation due to improved systolic left ventricular function

Cases are ordered based on timing of hospitalization for CIED infection

*AB* Antibiotic therapy; *CIED* Cardiac implantable electronic device; *CNS* Coagulase-negative staphylococci; *TLE* Transvenous lead extraction

The median PADIT score in patients with a CIED infection in the first year after the procedure was 6 (IQR, 4–8). The PADIT score showed good discrimination in predicting CIED infection requiring hospitalization within the first year (*C*-statistic 0.70; 95% CI 0.54–0.86, *P* = 0.03). The optimal cut-off was a PADIT score of ≥ 7 resulting in a sensitivity of 50% and a specificity of 83% for predicting CIED infection. Patients with a PADIT score ≥ 7 had a higher risk of hospitalization for CIED infection within the first year than patients with a lower PADIT score (1.23% vs. 0.26%, *P* = 0.02; odds ratio 4.8, 95% CI 1.4–16.6, *P* = 0.01).

In the 1117 patients who can be considered potential WRAP-IT candidates (i.e., CIED reoperations and initial CRT-D implantation) the incidence of CIED infection within the first year after the procedure was 0.45% (95% CI 0.15–1.04%).

Of the study population, a total of 130 patients (5.6%) died within 1 year of the procedure (cardiovascular death 32%, non-cardiovascular death 25%, unknown cause 44%). None of these 130 patients had a hospitalization for CIED infection.

## Discussion

The present study demonstrates that the risk of device infection can be low (0.43%) when strict adherence to preventive measures for CIED infections is used. The PADIT score was useful in identifying patients at high risk of CIED infection. For our tertiary referral center, a PADIT score ≥ 7 had the highest sensitivity and specificity for predicting CIED infection requiring hospitalization with a 1-year risk of 1.23%. Identification of this high-risk population for CIED infection is useful because they can potentially benefit from adjunctive preventive measures like an antibiotic envelope.

### Risk of CIED infection

CIED infection is associated with significant morbidity, increased hospitalizations, reduced survival, and financial health care burden [12]. The large prospective PADIT trial (*n* = 19,603) demonstrated a 1-year infection rate of 0.9% (2). It is important to note that most patients in the PADIT trial were high-risk patients (66%) who underwent either CIED reoperation or a CRT procedure. Infection risk is dependent on several patient-related, procedure-related, and device-related factors [17]. Important preventive measures to reduce the risk of CIED infections are the use of antibiotic prophylaxis, chlorhexidine skin preparation, delaying the procedure in case of fever, avoidance of heparin bridging, avoidance of pocket hematoma, the use of strict sterile techniques, and having experienced operators. These preventive measures are summarized in the 2019 EHRA international consensus document [16]. In comparison to the PADIT study population, our study population was younger (61 vs. 72 years), had a higher proportion of immunocompromised patients (5.4% vs. 1.6%), and had relatively more ICD implantation/replacement (42.6% vs. 21.6%). All these factors are independent predictors of a higher risk of CIED infection. However, the 1-year risk of CIED infection was low (0.43%, 95% CI 0.21–0.79%). Our results agree with a recent prospective single-center study using a real-world cohort (median age 77 years, median PADIT score 2 [IQR, 2–4]) which demonstrated a 1-year risk of CIED infection requiring hospitalization of 0.36% [18]. Thus, it seems that strict adherence to preventive measures may result in CIED infection rates well below 1% in an all-comer population.

### Identification of the high-risk patient

The WRAP-IT study demonstrated that an absorbable antibiotic envelope (TYRX™, Medtronic, MN, USA) reduced the risk of major CIED infection by 40% in high-risk patients [11]. It is important to realize that immunocompromised patients, patients with previous CIED infection, and hemodialysis patients were excluded in WRAP-IT. Cost-effectiveness studies in the USA and European health care systems demonstrated that the antibiotic envelope was cost-effective when the standard-of-care infection risk was ≥ 1.0% [12] or when the PADIT score was ≥ 6 [7]. The 2019 EHRA international consensus document recommends an antibiotic envelope in patients aligned with the WRAP-IT study population or other high-risk factors, in the context of the local incidence of CIED infections [16]. This last aspect is important to note because different centers have different standard-of-care infection rates depending on their patient populations and local preventive measures. Risk stratification with risk score calculators could play a useful role by providing an objective way to identify high-risk patients [14, 19, 20]. Such a calculator should be easy to use and be readily available for widespread adoption in clinical practice. We chose the PADIT score calculator [14], as this score has been validated in several independent cohorts supporting the generalizability of its use with a *C*-statistic ranging between 0.63 and 0.76 [3, 21, 22]. Besides the US Health claims database study, the number of patients in these validation cohorts ranged from 1000 to 2675 patients. In our study population (*n* = 2333), the PADIT score also provided good discriminative ability with a *C*-statistic of 0.70. Patients with a PADIT score of ≥ 7 comprised 17.4% of our study population and had a 1-year standard-of-care infection rate of 1.23%. This infection rate (≥ 1%) seems to justify an antibiotic envelope based on cost-effectiveness studies [12].

### Clinical implications

The different cost-effectiveness studies evaluating incremental cost-effectiveness ratios of the TYRX™ envelope used different costs of the antibacterial envelope depending on the specific country (USA, $669; Germany, €945; Italy, €945; England, £800) [7, 12]. Currently, there is no reimbursement for the antibacterial envelope in the Netherlands. Almost half of our study population fulfilled the inclusion criteria for WRAP-IT; however, the 1-year standard-of-care infection rate was < 0.5% in this specific cohort. This renders the use of an antibacterial envelope less cost-effective for our patient population based on WRAP-IT inclusion criteria. Restricting the use of antibacterial envelopes to high-risk patients according to the PADIT score (≥ 7) will be more cost-effective in our tertiary center because less than 20% of the patients will require an antibacterial envelope. Prospective randomized data should evaluate whether patient selection for an antibacterial envelope based on a high PADIT score (including clinical and procedural factors) is more cost-effective in comparison to using the eligibility criteria for WRAP-IT (mainly based on type of procedure).

### Study limitations

The present study has the known limitations inherent to a retrospective study design. Despite the retrospective study design, the variables needed for the PADIT calculator were readily available from the medical records. Furthermore, the primary endpoint comprised hospitalization for CIED infection which is an event that is usually well documented. The fact that our center is a regional endocarditis and lead extraction tertiary referral center reduces the risk of missing a clinically relevant endpoint. We did not focus on minor CIED infections not requiring hospitalization; this may explain the discrepancy in infection rates with other studies. Considering the single-center design and low number of events, the results of our study should be interpreted with caution, and larger prospective studies are warranted.

## Conclusions

When using strict preventive measures, the risk of CIED infection can be relatively low. The PADIT score had a good discriminative value in our study population for identifying patients at high risk for CIED infections. In our real-world all-comers cohort, a PADIT score of ≥ 7 identified a high-risk population in which an antibacterial envelope may be cost-effective considering a standard-of-care infection rate of > 1%. However, randomized trials are needed to evaluate whether the strategy of using an antibacterial envelope only in patients with a high PADIT score is beneficial and cost-effective.

## Supplementary Information

Below is the link to the electronic supplementary material.

Supplementary file1 (DOCX 16 KB)
