# Supplementary material for: Device infection in patients undergoing pacemaker or defibrillator surgery: risk stratification using the PADIT score
Source: J Interv Card Electrophysiol. 2024 Jan 30;67(6):1419–26. doi: 10.1007/s10840-024-01759-1 (PMC11379746; doi:10.1007/s10840-024-01759-1)
Supplement: Supplementary file 1 — Supplementary file1 (DOCX 16 KB) [file 10840_2024_1759_MOESM1_ESM.docx]

**Supplemental material**

**Supplemental table 1** – The PADIT risk score

| **Risk factor** | **Points** |
| --- | --- |
| 1. **P**rior procedure(s) on the same pocket    - 0    - 1    - ≥2 | 0  1  3 |
| 1. **A**ge    1. <60 years old    2. 60-69 years old    3. ≥70 years old | 2  1  0 |
| 1. **D**epressed renal function    1. Yes (eGFR <30 mL/min)    2. No | 1  0 |
| 1. **I**mmunocompromised    1. Yes (receiving therapy that suppresses resistance to infection or has a disease that is sufficiently advanced to suppress resistance to infection)    2. No | 3  0 |
| 1. **T**ype of procedure    1. Pacemaker: new or generator change    2. ICD: new or generator change    3. CRT: new or generator change    4. Revision or upgrade: pocket and/or lead revision and/or system upgrade, i.e., with adding new lead(s) | 0  2  4  4 |

Abbreviations: CRT, cardiac resynchronization therapy; eGFR, estimated glomerular filtration rate; ICD, implantable cardioverter-defibrillator. Source: https://padit-calculator.ca/
